# The Societal Impact of Herpes Zoster and Postherpetic Neuralgia on Patients, Life Partners, and Children of Patients in Germany

**DOI:** 10.1155/2014/749698

**Published:** 2014-12-08

**Authors:** Thomas Weinke, Andrea Glogger, Isabelle Bertrand, Kati Lukas

**Affiliations:** ^1^Klinikum Ernst von Bergmann, Gastroenterology and Infectious Disease, Charlottenstr 72, 14467 Potsdam, Germany; ^2^GfK Healthcare, Nordwestring 101, 90419 Nurnberg, Germany; ^3^Sanofi Pasteur MSD, 162 Avenue Jean Jaurès, CS 50712, 69367 Lyon Cedex 07, France

## Abstract

The aim of this study was to assess the impact of herpes zoster (HZ) and postherpetic neuralgia (PHN) on the daily activities of patients and family members who care for them. Some former patients and family members participated in face-to-face interviews or in a T-group meeting (qualitative phase) and some participated in telephone interviews (quantitative phase). They all expressed feelings of helplessness and frustration mixed with depression, sadness, or rage. Many of the former patients said their lives stopped, in contrast to family members who said that their lives were busy and stressful. Family members caring for patients with PHN were more psychologically stressed than those caring for patients with HZ. Although former patients appreciated the psychological and emotional support given by their family members, they underestimated the impact that their disease had on them. Former patients and their family never forgot this illness and its considerable impact on their lives, particularly when PHN occurred. We need to raise the awareness of the general public about the real life impact of HZ and PHN and their often severe, debilitating consequences and the potential benefits from vaccination.

## 1. Introduction

Herpes zoster (HZ) or shingles is the clinical manifestation of the reactivation of latent varicella zoster virus (VZV) which can occur several decades after the initial infection with varicella virus (chickenpox) [1]. Most adults have had chickenpox, most of the time in their childhood; therefore, almost everyone is potentially at risk of developing HZ. Following the initial infection, the virus becomes latent in the nervous system and when the virus is reactivated, the individual develops HZ. HZ is generally characterised by a unilateral vesicular rash, often with acute pain [2]. The risk of VZV reactivation and, therefore, HZ increases with age. Even if the reasons are not completely understood, a decline in VZV-specific immunity, observed with natural immunosenescence that occurs with aging or immunosuppression secondary to certain diseases or immunosuppressant therapy, is known to favour symptomatic reactivation of VZV [2]. The clinical course of HZ can involve major complications, such as postherpetic neuralgia (PHN) which is a frequent, debilitating complication [3]. Although there is no consensus on the standard definition for PHN, it is often defined as pain that persists for ≥3 months after the onset of the HZ rash [4]. There are no factors that can predict who will develop HZ or how severe the disease will be. In this setting of unpredictability of the disease and its severe consequences, prevention is important. Currently, there are no preventative drug treatments for HZ or PHN, and although some treatments are available, pain control is often difficult and unsatisfactory. In this setting, the aim of HZ treatment is to limit viral replication at an early stage of the disease and to relieve pain but it cannot prevent the onset of PHN. There is an unmet medical need [5]. The only effective prevention strategy is vaccination.

Increasingly, the importance of quality of life (QoL) and other patient-reported outcomes has been recognised in many disease areas, particularly in the absence of clinical data. Several observational studies have reported the significant impact that HZ and PHN can have on the patients' QoL and their daily activities [6–10]. These conditions can also have a deleterious effect on the patients' functional ability, even resulting in patients becoming housebound or inactive, with a greater impact on those who develop PHN [10–12]. While many studies have assessed the impact of HZ and PHN on the QoL and daily activities of patients, to our knowledge none have assessed the broader societal impact of the disease on the relatives of patients with HZ or PHN. The real impact of HZ and PHN remains largely underestimated. This was illustrated in one study that included patients with and without HZ (recruited through general practitioners) and controls from the general public. The results showed that although the controls were aware of HZ and its associated pain, the level of knowledge of patients with HZ was higher [13]. This shows that there is a need to increase awareness.

The aim of our study was to assess and quantify the societal impact of HZ and PHN on patients and their family members (life partners or children) who were involved in caring for the patient using qualitative and quantitative methods and patient-reported outcome tools.

## 2. Methods

The study was designed with two phases: a qualitative and a quantitative phase using specifically designed questionnaires developed by a multidisciplinary team. More detailed information on the study design is available on request from the authors. The aim of the qualitative phase was to evaluate in detail the overall societal impact of HZ/PHN and its burden on patients and their relatives. The information gathered was used to formulate hypotheses that were tested in the quantitative phase.

The study was performed in subjects who had suffered from HZ or PHN during the previous five years. The patient was classified as HZ if the HZ-associated pain lasted <3 months after rash onset and as PHN if the HZ-associated pain lasted ≥3 months after rash onset. Both patients and family members were recruited through physicians (mainly general practitioners) to ensure that the patients had been correctly diagnosed with HZ or PHN by a physician.

### 2.1. Qualitative Phase

The subjects were invited to participate in either an in-depth face-to-face interview or a therapeutic group (T-group) interview. A psychologist conducted the T-group interview and together with a social scientist conducted the individual face-to-face interviews in a studio setting with a video recording that was later analysed. Both the psychologist and the social scientist were employees of GfK Healthcare who were contracted to conduct the study.

#### 2.1.1. Face-to-Face In-Depth Interviews

The screening criteria for the former HZ/PHN patients included age (50–59, ≥60 years), patients who had suffered from HZ/PHN in the previous five years, those who were aged ≥50 years when they suffered from HZ/PHN, and those who had consulted a physician because of their shingles. The former patients had had to have pain associated with the shingles. The screening criteria for the family members of former patients were that the former patient had to be aged ≥50 years, the life partners had to be aged ≥50 years, and children of former patients had to be aged between 20 and 49 years.

There were 12 face-to-face in-depth interviews conducted in Düsseldorf and Frankfurt, which lasted for 75 minutes with

- three former patients who suffered from HZ;
- three former patients who suffered from PHN;
- three life partners of former patients;
- three children of former patients who lived close to their parent.

The areas covered during these interviews were

- their rational and emotional perception of HZ or PHN;
- the emotional and rational challenges of their life with HZ or PHN or of living with a patient;
- detailed assessment of the acute versus chronic stages: particularly for the impact on their close and more distant social environment; functional impact including routine daily activities such as work or housework and other physical activities; expenses incurred, in addition to those reimbursed by their health insurance; psychological impact;
- summary of their experience with HZ or PHN and their outlook for the future.

#### 2.1.2. Therapeutic Group (T-Group)

**Figure 1:**
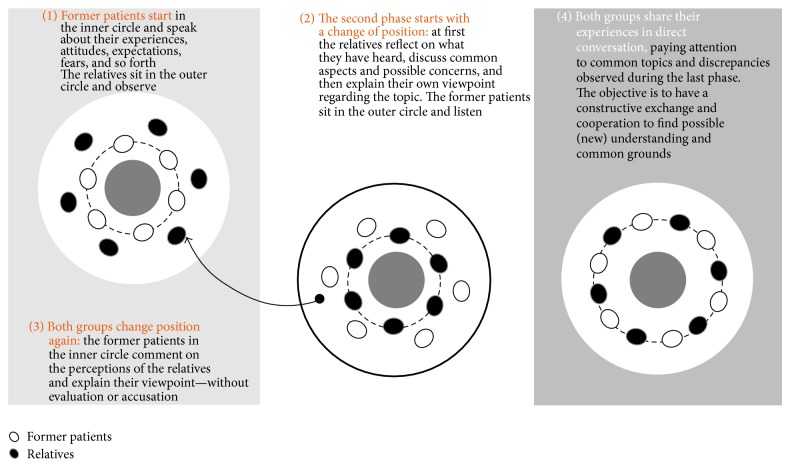
Outline of the T-group process used in the qualitative survey.

The so-called T-group methodology was originally developed in psychology [14]. Since then it has been used very successfully in qualitative market research (Figure 1). The T-group methodology allows two different target groups, in our case, former patients and family members that are usually not brought together, to be questioned at the same time. Although homogeneous groups have greater cohesion, heterogeneity and diversity within a group can be an asset as this provides the participants with multiple knowledge bases and different perspectives to help the group members to understand others points of view. Thus, it is critical that group cohesion be a focus of heterogeneous groups so that the multiple perspectives are respected and supported [15–17].

Homogeneity is present within the subgroups and heterogeneity between the two target groups. The openness of the patients and family members is strengthened through the group setting as they feel accepted and understood in their subgroups and are backed-up by their peers. In contrast with a standard group setting, it is even more important to establish an atmosphere of trust, warmth, and empathy as well as understanding and acceptance. In this context, the homogeneity of the subgroups is motivational and contributes to the group cohesion which is essential to maximize the output, particularly given the time constraints.

In contrast to long-term group therapy, the patients and family members who participated in this short-term study design were not related. If they had been related, the short-term setting would not allow enough time and space to address the personal history of the participants' relationships satisfactorily. Additionally, when designing this approach for this project, we decided to include former patients and not current patients who would still be suffering and could find the interview setting uncomfortable. Each individual could state their perception so that similarities, differences, and contradictions were observed. The mutual exchange of perspectives, first in the subgroups and then in the whole group, moderated by a psychologist can make it possible to talk about even very sensitive topics. Combining the in-depth interviews with a group setting enabled us to obtain an overall assessment of the situation, which in our case was an understanding of the patient-family interaction and the societal impact of HZ and PHN.

The way the discussions are structured and moderated is important for the success of this approach. For much of the session, the two groups were questioned separately (Figure 1). This enabled the former patients to express themselves first without being influenced by the family members, while the family members were encouraged to listen and understand. After this, the entire group discussed what they had learnt and understood. The group of family members could then talk about their perceptions in a “protected” setting while the former patients were asked to listen and to take notes without interrupting the family members. To help the group to focus on either one subgroup or the entire group, we physically separated or mixed them. There were no tables in the room and the participants sat on chairs arranged in two circles. If the questions were targeted at a subgroup, the other moved to the outer-circle (Figure 1) and if the whole group was involved the members of the subgroups were mixed. One of the moderators always stayed with one of the subgroups.

The T-Group interviews were conducted with five former patients (one had had HZ and four had had PHN) and five former-patients' family members (unrelated to the former patients in the group). The “family members” were either life partners or children who lived close to former patients. The interviews lasted 240 minutes in a studio setting in Berlin, Germany, with video recording for later analysis. The areas covered during these interviews were as follows:

- with former patients: their general perception of HZ or PHN and how they lived with the disease;
- with family members: their experience of the disease with the former HZ or PHN patients and how they lived with them during the illness;
- with former patients: detailed assessment of the individual stages of the disease;
- with family members: reactions to the detailed assessment of individual stages, with the main objective of encouraging a direct exchange of perspectives about their situation, their understanding of the underlying emotions, the impact the disease had on their daily lives, and any changes in their QoL.

### 2.2. Quantitative Phase

#### 2.2.1. Study Design for Telephone Survey

The telephone survey was undertaken using the in-house telephone studio of GfK Healthcare (who performed the survey). The interviewers were native-German speakers with extensive experience in healthcare surveys. The survey, which was carried out between October 14, 2009, and November 16, 2009, involved a 20-minute interview with specifically designed questions that covered various dimensions as follows.

Overall dimensions explored during the 20-minute telephone interview with former patients who had had herpes zoster or postherpetic neuralgia and family members of former patients the following:

1. impact on social, psychological, health, and financial aspects:
  - perceived quality of life,
  - daily living activities,
  - family life,
  - relationship with life partner,
  - social life (seeing friends and doing hobbies and leisure activities),
  - psychological life (stress, fatigue, insomnia, and psychological impairment),
  - health status (depression or anxiety, weight loss, and use of medication),
  - financial impact;
2. impact on professional life:
  - time off work or change to flexible working hours (for relatives),
  - length of absence from work (for former patients).

The former patients were recruited via their physician and were representative of the German population aged over 50 years in terms of the region where they lived and their household income.

The sample was composed of 168 former patients (HZ: *n* = 114; PHN: *n* = 54) who had consulted their physician for their disease and who had had associated pain and 162 family members (life partners: *n* = 95; children: *n* = 67) (Table 1). To be eligible, the former patients had to have been ≥50 years old when they suffered from HZ (<3 months of pain) or PHN (≥3 months of pain) in the five years prior to the survey. The children of the former patients, who had to live near them, were mostly aged 20–49 years. The life partners of the former patients were mostly aged ≥50 years.

## 3. Results

### 3.1. Qualitative Study Results

The results showed that HZ and PHN generally have a high impact on the lives of both the former patients and the family members. The majority of the former patients and family members perceived the disease as an important, crucial event in their lives. The former patients' main impressions about the disease were the pain and feeling physically “knocked out.” There were strong feelings of helplessness and frustration mixed with depression, sadness, or rage for both the former patients and the family members. Many of the former patients said their lives came to virtual standstill in terms of daily activities, hobbies, leisure activities, and social life; they expressed a feeling of “deadlock.” This was in contrast with the family members who said that their lives became very busy and sometimes stressful. There seemed to be a relatively low impact on the professional lives of the former patients who were working while they were sick (mainly sick leave); the impact on the professional lives of family members was also relatively low, with only a few taking holiday leave or changing working hours. The main societal burden of HZ and PHN seemed to be human rather than economic and the reported costs incurred were predominantly personal nonmonetary costs. The new role for the “carers” and the former patients' behavioural changes were reported to be sources of stress in their relationships. The former patients and relatives were worried and feared the torment, obsession, and distress caused by the additional burden on their relationships.

Generally, awareness about HZ and PHN was low, although some patients and relatives knew something about the disease aetiology. They said that they knew that triggers such as stress, psychological factors, or a weak immune system could reactivate the chickenpox virus “from childhood” that was “encapsulated” in the body and this reactivation could cause rash and pain. Most of the participants were unsure if the disease was contagious and what the chances of reoccurrence were. They reported a change of attitude towards the disease over time (a before-after phenomenon); usually before, HZ and PHN were considered as “harmless,” whereas after they were considered as “severe or very severe.”

Following this analysis, we identified the following statements which we used to develop a hypothesis that was verified in the second, quantitative part of the study:

- HZ and PHN are perceived as a sign of ageing, “becoming really old”;
- HZ and PHN are part of the comorbidities associated with ageing, a sign of decreasing immunity;
- families and friends volunteer to help and help to keep the moral up;
- the longer the condition lasts the harder it is for patients and families and friends to cope.

### 3.2. Quantitative Study Results

Many former HZ/PHN patients were unable to leave their house or even get up from their sofa. More than 85% of patients with HZ/PHN said that the disease had a moderate to high impact on their daily life and more than 60% of them said the disease had a moderate to high impact on their family life. In addition, about 40% of them said there was a moderate to high impact on their professional life and their relationship with their partner.

The majority of the former patients (HZ = 70%; PHN = 76%) had to have a carer who was most frequently a life-partner (50%) and/or a child (30%), followed by a friend or neighbour. Only very few patients had professional, paid carers. The majority of life partners had cared for the former patient alone, whereas the majority of the former patients' children had support from their other parent, their own life partner, or their own children. The family members of former HZ patients said they had to give support for about one month, compared with three to four months for those of former PHN patients. They said the main areas of support were doing shopping and housework and visiting physicians with the former patient, followed by psychological and emotional support (Table 2). The former PHN patients assessed the psychological and emotional support as being the most important (Table 2).

The majority of family members (69% children; 80% life partners) of patients with HZ or PHN said that caring for the patient resulted in a moderate to severe impact on their life. Most of them said that the patient's disease had caused them to suffer daily or several times a week from fatigue (life partners: 73%; children: 62%), stress (life partners: 66%; children: 68%), insomnia (life partners: 57%), and emotional distress (life partners: 56%; children: 62%). The family members of former PHN patients (>60%) said they felt significantly more psychologically impaired than those of former HZ patients did. The most negative aspect of the family members' self-assessed QoL was lack of time; 2/3 said they felt moderately to highly affected by having less time for going out with friends, leisure activities, and their own family.

Family members mainly took vacation or organised flexible working hours to care for the former patient. Life partners had to miss work or change to flexible working hours more often than children (42% versus 20%) to care for the former patient. The family members took time off work most often to attend healthcare visits with the former patient. The range of time was from an average of 15 times for part of a day, up to an average of seven whole days. About 40% said they had suffered from high mental pressure and stress at work due to taking time off work.

The former patients underestimated the psychological and social impacts of their illness on their life partner's or child's health and professional lives. Although more than 60% of the patients with HZ or PHN said that their disease had a moderate to high impact on their family life, their evaluation of the impact was lower than that of the family members' self-assessment (Table 3). In addition, the impact of the disease on the normal life of life partners and children was greater for those caring for patients with PHN than those caring for patients with HZ.

Children of patients with PHN reported significantly more depression than that assessed by the former patients (34% versus 16%). Children of patients with PHN and life partners of both patients with HZ and those with PHN reported a significantly higher impact of emotional distress, stress and fatigue, and insomnia than children of patients with HZ. Partners and relatives of patients with PHN (>60%) felt significantly more psychologically impaired than children or relatives of patients with HZ (63% versus 53%; *P* = 0.05).

## 4. Discussion

Many studies have assessed the impact of HZ/PHN on the patients' lives but, to our knowledge, this is the first study to assess the impact on those who care for these patients, as well as the patients [6–10, 12, 18, 19]. Our results show that an important impact of HZ and PHN on the lives of patients and their family members (life partners and children) is hidden. In addition, the patients themselves seem to underestimate this impact on the life partners and children.

In the qualitative study, we observed that knowledge about the aetiology of HZ and PHN was poor and that the disease was perceived as mild, prior to having direct (former patients) or indirect (family members) experience of the disease. The perception that HZ was a mild disease by those who were not affected was also reported in a study in Spain on patients with and without HZ and healthy individuals [13].

One of the advantages of our study was that in the first part we used two different methodologies to obtain our qualitative results, which were confirmed in the second part with a quantitative approach, with a larger sample. Also, this study is the first to assess the impact of HZ and PHN on both former patients and family members involved in the care of these patients, in the same study. This approach, which is new in social research, has allowed us to develop a broader picture by combining both internal (patient) and external (family members/carers) experiences.

One of the limitations of our study was the relatively small sample size in the qualitative survey. Although small sample sizes are generally acceptable for this type of methodology, larger-scale studies are needed to evaluate if the results can be extrapolated. Our inclusion criteria required that patients had to have HZ with pain and while almost 90% of patients have pain, the remainder do not [20–25]. This inclusion criterion could have led to the selection of patients with a more severe disease. In addition, although the qualitative phase of the study was performed in three towns, they were all located in Germany; therefore, the study should be repeated in other countries to confirm the results.

A live-attenuated VZV vaccine (Zostavax) has been developed with the objective of reducing the incidence and severity of HZ and its complications, particularly PHN, in adults aged 50 years and more. This vaccine is a paradigm shift in infectious disease management as it is the first licensed vaccine to prevent disease in patients already infected with the pathogen (VZV). By boosting the VZV-specific cellular immunity, the vaccine controls both reactivation and replication of the latent virus to reduce the burden of disease. Its clinical efficacy has been assessed in two pivotal placebo-controlled, double-blind clinical trials, the Shingles Prevention Study (SPS) and ZEST [26, 27]. Its efficacy was assessed in terms of reducing the burden of illness (BOI), that is, the incidence, severity, and duration of HZ-related pain and discomfort, the incidence of HZ, and the incidence of PHN. A reduction of 66% in the interference with activities of daily living was also reported [28]. Its clinical benefit and good safety profile have also been confirmed in real life conditions [29–32].

The VZV vaccine (Zostavax) was recommended in individuals aged ≥60 years in the US and Canada in 2007 and 2010, respectively. This vaccine was licensed in Europe since 2006 and some European countries, including the UK, Austria, Saxony (Germany), and Sweden, have recently decided to recommend and/or fund this vaccination. Our results suggest that the general public's knowledge about the disease and its consequences could have an important role for the acceptance of the vaccine and therefore the success of a vaccination programme. The study participants suggested that it would be difficult to be interested in the vaccine before the disease occurred, since the disease is perceived as mild, and when the disease occurs and perceptions change, it is too late to vaccinate. It is possible that an educational programme is needed to inform the general public about the disease and its consequences and about the benefits of vaccination. Previous studies have shown that the general public rely on their general practitioner for information and advice about vaccines, particularly new vaccines [33–35]. General practitioners and other health professionals involved in the care of adults ≥50 years could play a central role in patient education and therefore in the success of HZ vaccination.

## 5. Conclusions

In this study, we showed that HZ and PHN had a significant impact, not only on the lives of patients, but also on the lives of the family members who cared for them during the illness. The life partners and children of former patients who had PHN felt more psychologically impaired than those of patients who had HZ. Experience of HZ or PHN, either as a patient or as someone caring for a patient, changed the perception of the nature of the disease, particularly its severity. Former patients and their family never forget this condition and its considerable impact on their lives, particularly if PHN occurs. The patients themselves generally underestimated the impact of their disease on their life partners and children who cared for them. We need to raise general public awareness about HZ and PHN and their often severe, debilitating consequences and about the potential benefits from vaccination.

## Figures and Tables

**Table:** (a) Former patients with herpes zoster or postherpetic neuralgia

Characteristic	Herpes zoster	Postherpetic neuralgia	Overall
	(*n* = 114)	(*n* = 54)	(*n* = 168)
Male/females (*n*)	26/88	18/36	44/124
Mean age (years)	62.5	64.2	63.0
Age group (*n*)			
50–59 years	56	21	77
≥60 years	58	33	91

**Table:** (b) Family members of former patients with herpes zoster or postherpetic neuralgia

	Life partner	Child	Overall
	(*n* = 95)	(*n* = 67)	(*n* = 162)
Male/female (*n*)	53/42	14/53	67/95
Age group (*n*)			
20–49 years	4	52	56
≥50 years	91	15	106
Related to former patient			
Herpes zoster	61	48	109
Postherpetic neuralgia	34	19	53

**Table 2:** Perceived importance of the different areas of support by the carers (life partner or child) and former HZ and PHN patients. The data for the former patients is given as a function of who cared for them, either life-partner or child. The data are the percentages of participants who said the area of support was important.

*Question asked: What does your carer (life partner or child) do for you? *						
Area of support	Life-partners	Children	Former HZ patients		Former PHN patients	
			Life-partners	Children	Life-partners	Children

Shopping	83	84	86	81	71	40
Housework	80	75	70	66	74	60
Visiting physician	66	63	64	66	68	27
Psychological/emotional support	47	36	55	56	77	80
Basic activities	34	28	39	25	47	27
Washing/showering	27	30	20	28	35	20

**Table 3:** Percentage of respondents who replied ≥4 (on a scale of 0 (none) to 10 (high)) to questions about the impact of the disease on the different aspects indicated. The former patients gave their assessment of the impact for their carer; the carers gave their self-assessment.

*Question asked: Caring for or supporting another person that is ill may have an impact on your own health. On a scale from 1 to 10, 1 meaning “does not apply at all” and 10 meaning “applies fully”, tell me to what extent the following areas were impacted when took care of or were supporting your (life-time partner/parent) during their shingles. *							
	Fatigue	Weight loss	Insomnia	Emotional distress	Psychological impairment	Depression/anxiety	Stress

Patients assessment for their carer							
HZ patients	38	12	27	47	31	19	53
PHN patients	36	4	27	39	29	16	39

Self-assessment by the carer							
Life partners	63	14	46	66	51	30	64
Children	45	13	30	49	34	21	60
Relatives of HZ patients	52	14	35	54	41	22	59
Relatives of PHN patients	60	13	49	70	49	39	70
